# Case report: Complete resection of invasive thymoma invading the superior vena cava and right atrium under cardiopulmonary bypass support

**DOI:** 10.3389/fonc.2022.1026524

**Published:** 2022-10-20

**Authors:** Xiangxin Zhang, Liang Chen, Wenyong Zhou, Zhexin Wang, Chong Wang, Jianxin Shi, Feng Yao

**Affiliations:** ^1^ Department of Thoracic Surgery, Shanghai Chest Hospital, Shanghai Jiao Tong University School of Medicine, Shanghai, China; ^2^ Department of Emergency Medicine, Qilu Hospital of Shandong University, Jinan, China; ^3^ Shandong Provincial Clinical Research Center for Emergency and Critical Care Medicine, Institute of Emergency and Critical Care Medicine of Shandong University, Chest Pain Center; Qilu Hospital of Shandong University, Jinan, China; ^4^ Key Laboratory of Emergency and Critical Care Medicine of Shandong Province, Key Laboratory of Cardiopulmonary Cerebral Resuscitation Research of Shandong Province, Shandong Provincial Engineering Laboratory for Emergency and Critical Care Medicine, Qilu Hospital of Shandong University, Jinan, China; ^5^ The Key Laboratory of Cardiovascular Remodeling and Function Research, Chinese Ministry of Education, Chinese Ministry of Health and Chinese Academy of Medical Sciences, The State and Shandong Province Joint Key Laboratory of Translational Cardiovascular Medicine, Qilu Hospital of Shandong University, Jinan, China

**Keywords:** thymoma, invading the right atrium, cardiopulmonary bypass, surgery, case report

## Abstract

Here we describe an uncommon case of a 48-year-old male patient with an invasive thymoma invading the superior vena cava, bilateral innominate veins, right internal jugular vein, right subclavian vein, right atrium, azygos vein, and part of the lung tissues. The tumor was resected entirely under cardiopulmonary bypass support, and the venous bypass using a vascular graft was successfully established between the left innominate vein and the right atrium. The postoperative course was uneventful, and the patient was discharged 15 days after surgery without complications.

## Introduction

Thymoma is the most common primary anterior mediastinal tumor in adults (1), which can be further classified into the invasive or non-invasive type according to its extracapsular extension (2). For invasive thymoma, the surgical treatment often involves adjacent resectioning structures, such as either of the innominate veins, superior vena cava (SVC), pericardium, pleura, and the lung. The resection and reconstruction of the SVC system are considered feasible (3, 4), but it remains unclear when the SVC system and the right atrium are involved.

Here, we report a case of an invasive thymoma completely resected under cardiopulmonary bypass (CPB) support, which invades the SVC and the right atrium together with the bilateral innominate veins, the right internal jugular vein, the right subclavian vein, the azygos vein, and part of lung tissues.

## Case report

**Figure 1:**
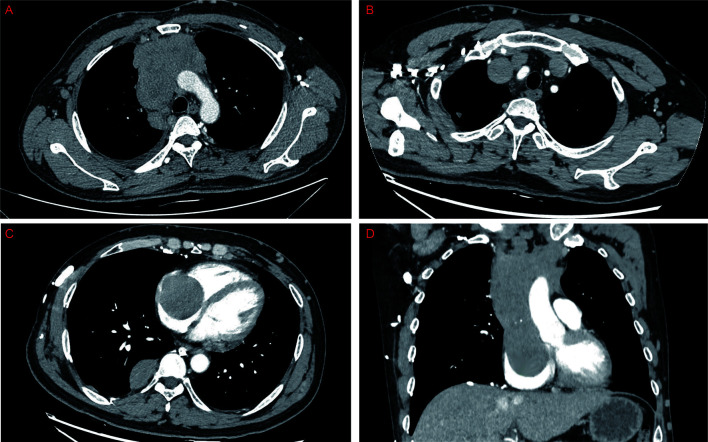
Preoperative computed tomography showed that the thymoma located in the anterior mediastinum **(A)** invaded the bilateral innominate veins, the superior vena cava, and the right atrium **(B–D)**.

A 48-year-old male patient was admitted to our center with the complaint of chest tightness. Computed tomography demonstrated a mass in the anterior mediastinum, measuring 13 cm × 7 cm, which was suspected of invading the SVC and the right atrium (**Figure 1**). Positron emission tomography/computed tomography scan showed no distant metastasis but mediastinal lymph node metastasis. According to the fine-needle aspiration biopsy results, the mass was considered to be type B2 thymoma. As the multidisciplinary team suggested, the patient was treated with three-cycle chemotherapy with cisplatin, cyclophosphamide, and epirubicin hydrochloride, followed by oral prednisolone for 1 month. However, the response was finally assessed as a stable disease by the Response Evaluation Criteria in Solid Tumors (version 1.1) (5). The patient had no myasthenia gravis but had mild SVC syndrome (6), so surgical treatment was decided.

**Figure 2:**
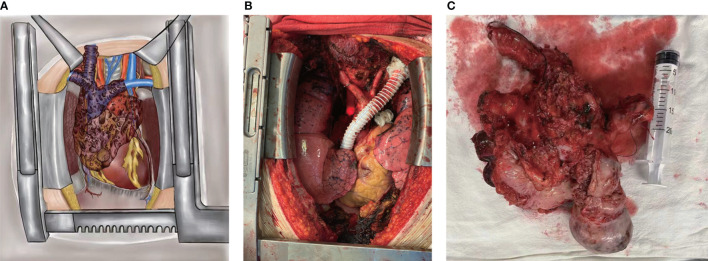
During the operation, the tumor was observed to invade the superior vena cava and the right atrium together with the bilateral innominate veins, the right internal jugular vein, the right subclavian vein, the azygos vein, and the lung **(A)**. The tumor was completely resected, and the venous bypass was established between the left innominate vein and the right atrium **(B)**. Tumor specimen **(C)**.

Through a median sternotomy, the tumor in the anterior mediastinum was observed to invade the SVC and the right atrium together with the bilateral innominate veins, the right internal jugular vein, the right subclavian vein, the azygos vein, and the lung (**Figure 2A**). The left innominate vein was transected near the left venous angle and then anastomosed with the vascular prosthesis. To establish the CPB, the arterial cannula was inserted into the right femoral artery, whereas the venous cannulas in a bicaval fashion were respectively inserted into the right femoral vein and the vascular prosthesis. After that, the right internal jugular vein, the right subclavian vein, and the azygos vein were all ligated and cut off, and wedge resection for part of the lung tissues was performed using the linear staplers. Then, the mass was separated from the aorta arch and right hilum. Through a longitudinal incision of the anterior wall of the right atrium, the tumor was detected to invade part of the right atrium around the entrance of the SVC. Therefore, the right atrium was partially resected, and the tumor was removed entirely, followed by the venous bypass reconstruction between the left innominate vein and the right atrium (**Figures 2B, C**). During the operation, the sinus node and the left phrenic nerve were both preserved, whereas the right phrenic nerve was also resected due to tumor invasion. At the end of the surgery, the CPB was safely weaned off. The internal jugular vein pressure was below 20 cmH_2_O, and the patient was successfully extubated in the operating room.

**Figure 3:**
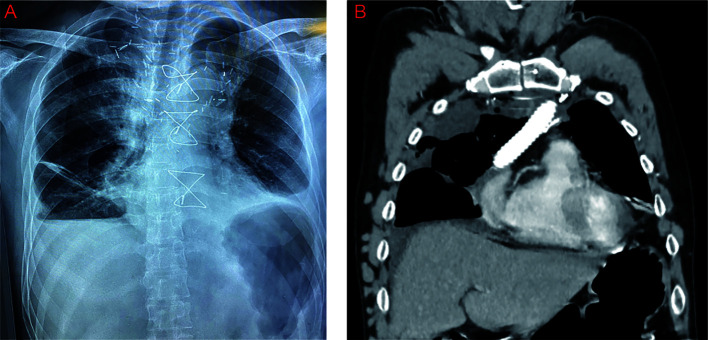
Postoperative X-ray **(A)** and computed tomography **(B)** imaging of the patient.

The final histopathological examination confirmed the type B2/B3 mixed-type thymoma (2021 WHO Classification) and negative resected margins (Masaoka–Koga stage IIIB, T4N0M0) (7–9). The postoperative course was uneventful, and the patient was discharged 15 days after surgery without any complications (**Figure 3**). In order to detect the patient’s blood coagulation status and physical condition, the current follow-up interval is 1 month. The patient will be treated with adjuvant chemotherapy after surgery.

## Discussion

Thymoma invading the great mediastinal vessels and the right atrium simultaneously is extremely rare. Several cases have potentially revealed the promising prognoses of patients receiving surgical resection for invasive thymomas compared with those who underwent the non-surgical treatment (10–13). Kumar et al. reported the survival outcomes in 12 patients receiving surgical resection for locally advanced thymoma with SVC invasion. The 1-, 3-, and 5-year overall survival rates were 100%, 91.6%, and 83.3% in their cohort, respectively (14). After the literature review, Kurata et al. summarized the survival outcomes in 23 cases of thymoma invading the atrium. Among 20 patients receiving surgical treatment, three died within 1 month, at 2 months, and 1 year after surgery. The average survival time of the other 17 patients was 30.1 months, and the longest follow-up period was 8.5 years. However, all three remaining non-surgical treatment patients died during follow-up (15).

According to previous literatures, CBP has often been used in thoracic malignant tumors invading the heart or great vessels, trachea, or carina (16). Studies have shown that using CPB does not appear to increase the risk of tumor dissemination. For patients with locally advanced cancers, CBP can safely help remove the tumor and improve the survival to a certain extent (17, 18).

Complete resection of invasive thymoma and the involved structures (such as the great mediastinal vessels) would benefit the oncological prognosis (19). Traditionally, when the tumor invades SVC and the confluence of bilateral innominate veins, the “Y-graft” or “two separate grafts” is usually selected to reconstruct the venous drainage. When both SVC and the long segment of a single innominate vein are resected, a single straight graft between the uninvolved innominate vein and the right atrium is applied (14). In the present case, the SVC, bilateral innominate veins, the azygos vein, and part of the right atrium were all resected to perform complete resection. Meanwhile, the right internal jugular and right subclavian valves were also ligated at a high level, wherein establishing venous bypass between these vessels and the right atrium would be quite difficult (**Figure 2A**). Therefore, the venous drainage was only reconstructed between the left innominate vein and the right atrium. Even so, due to the development of the venous collateral circuits after the long-term occlusion of SVC, there were no clinical signs of SVC syndrome or elevated internal jugular vein pressure. Furthermore, the patient’s symptoms were relieved to a great extent in this case. It was also previously reported that the symptomatic patients who suffered thoracic malignancies invading the heart or great vessels had immediate and sustained palliation of their symptoms after surgical treatment (20).

In conclusion, radical resection for such an invasive thymoma may be safely attempted in selected patients under CPB support, which may help achieve prolonged survival.

## Data availability statement

The original contributions presented in the study are included in the article/supplementary material. Further inquiries can be directed to the corresponding authors.

## Ethics statement

The studies involving human participants were reviewed and approved by the ethical committee of the Shanghai Chest Hospital. The patients provided their written informed consent to participate in this study. Written informed consent was obtained from the individual(s) for the publication of any potentially identifiable images or data included in this article.

## Author contributions

FY, JS, WZ, CW and ZW performed the surgery together. XZ and LC wrote the manuscript. All authors contributed to the article and approved the submitted version.

## Acknowledgments

We are very grateful to the anesthesiology, surgical nursing, and intensive care unit team at Shanghai Chest Hospital for their efforts.

## Conflict of interest

The authors declare that the research was conducted in the absence of any commercial or financial relationships that could be construed as a potential conflict of interest.

## Publisher’s note

All claims expressed in this article are solely those of the authors and do not necessarily represent those of their affiliated organizations, or those of the publisher, the editors and the reviewers. Any product that may be evaluated in this article, or claim that may be made by its manufacturer, is not guaranteed or endorsed by the publisher.
